# Fukushima and Chernobyl: Similarities and Differences of Radiocesium Behavior in the Soil–Water Environment

**DOI:** 10.3390/toxics10100578

**Published:** 2022-09-30

**Authors:** Alexei Konoplev

**Affiliations:** Institute of Environmental Radioactivity, Fukushima University, 1 Kanayagawa, Fukushima 960-1296, Japan; r701@ipc.fukushima-u.ac.jp

**Keywords:** Fukushima, Chernobyl, NPP, radiocesium, hot particles, leaching, sorption, fixation, soil, water, environment

## Abstract

In the wake of Chernobyl and Fukushima accidents, radiocesium has become a radionuclide of most environmental concern. The ease with which this radionuclide moves through the environment and is taken up by plants and animals is governed by its chemical forms and site-specific environmental characteristics. Distinctions in climate and geomorphology, as well as ^137^Cs speciation in the fallout, result in differences in the migration rates of ^137^Cs in the environment and rates of its natural attenuation. In Fukushima areas, ^137^Cs was strongly bound to soil and sediment particles, with its bioavailability being reduced as a result. Up to 80% of the deposited ^137^Cs on the soil was reported to be incorporated in hot glassy particles (CsMPs) insoluble in water. Disintegration of these particles in the environment is much slower than that of Chernobyl-derived fuel particles. The higher annual precipitation and steep slopes in Fukushima-contaminated areas are conducive to higher erosion and higher total radiocesium wash-off. Among the common features in the ^137^Cs behavior in Chernobyl and Fukushima are a slow decrease in the ^137^Cs activity concentration in small, closed, and semi-closed lakes and its particular seasonal variations: increase in the summer and decrease in the winter.

## 1. Introduction

The disasters at the Chernobyl nuclear power plant (ChNPP) (USSR, April 1986) and Fukushima Daiichi nuclear power plant (FDNPP) (Japan, March 2011) are the only two nuclear accidents rated as level 7 by the INES (International Nuclear Event Scale) of the International Atomic Energy Agency (IAEA). Both accidents led to the large-scale radioactive contamination of the environment [1,2], and in both cases, the radionuclide of most significance for the environment, defining long-term consequences, is ^137^Cs with a half-life of 30.2 years [3,4].

The behavior of accidentally released radionuclides in the terrestrial and aquatic environment is contingent on the interrelationship of their chemical forms in the fallout and the characteristics of the environment determining the rates of their transformation and transport processes [5,6,7,8,9]. Of major importance are the geoclimatic characteristics of the contamination zone such as precipitation, mean annual air temperature, terrain, and land use [8,10]. The climate and geographical conditions for Fukushima Prefecture of Japan and the Chernobyl-contaminated areas of Ukraine, Belarus, and Russia display substantial differences. The catchments of the Chernobyl zone are flat and characterized by minor slopes, whereas Fukushima’s catchments are mainly hilly and have relatively steep slopes. Annual precipitation also markedly differs and is more than two times higher in the FDNPP area (1420 mm) [11] than at Chernobyl (625 mm) [8,9]. Moreover, in the Fukushima region, high flow events in rivers occur especially during the typhoon season, which is conducive for radionuclide wash-off from contaminated catchments and its lateral migration [12,13,14,15,16].

A large difference exists between the soils of the Fukushima- and Chernobyl-contaminated areas. The Chernobyl soils are made of outwash sands and alluvial deposits, mainly of loamy-sand composition containing a lower proportion of silty fraction as compared with the Fukushima region. Parent rock materials in Fukushima are primarily granites and volcanic ashes that are subject to physicochemical weathering in the humid monsoon climate conditions. The proportion of clays in Fukushima is 20–30% [17], which is higher than those in the sandy loam soils of the Chernobyl area, even though the sand fraction makes up 40–50% in both Fukushima and Chernobyl, on the average [18].

In addition to geoclimatic differences, the speciation of initially deposited ^137^Cs at Fukushima and Chernobyl differed [9,19]. Thus, the difference in the initial speciation of ^137^Cs in Chernobyl and Fukushima, as well as the distinctions in the composition of the soils, along with the dissimilarity in geoclimatic conditions, could be responsible for the differences in the radiocesium fate in the environment. The objective of this review was to analyze available data on the long-term behavior of Chernobyl- and Fukushima-derived radiocesium in a soil–water environment to identify qualitative and quantitative similarities and differences. It also aims to compare major governing factors and characteristics of the radiocesium fate and transport in the environment.

## 2. Speciation of Radiocesium and Its Transformation in Soil–Water Environment

### 2.1. Release of Hot Particles following Chernobyl and Fukushima Accidents

#### 2.1.1. Chernobyl

Owing to the explosion shock wave, temperature gradient, and oxidation of nuclear fuel, hot (highly radioactive) fuel particles were formed. The release of these fuel particles into the environment was the main distinguishing feature of the radioactive contamination following the Chernobyl accident [5,20,21,22,23,24,25]. The radionuclide composition of the fuel particles was similar to the fuel make-up in the damaged unit with some depletion of volatile nuclides (^131^I, ^134,137^Cs, ^106^Ru, etc.). Sizes of deposited fuel particles after the Chernobyl accident ranged from hundreds of microns to a fraction of a micron [23]. Within the 30 km zone of the Chernobyl NPP, up to 10^5^ particles/m^2^ have been observed [22]. Surface loading of fuel particles decreased with an increasing distance from the reactor site [23].

Due to the accident, considerable amounts of volatile fission products (I, Cs, and others) were released into the air and partly condensed on inert particle carriers [21,25]. The hot particles formed in this way had contaminated surfaces and were characterized by a lower specific activity compared with fuel particles [25,26]. Radiocesium compounds condensed on the surfaces of dust particles are normally soluble in water since Cs is an alkali element, and, therefore, they dissolve in raindrops in the atmosphere, soil solution, or water bodies. On the other hand, radiocesium incorporated in fuel particles is not available for direct exchange with water and dissolution. It is nonexchangeable, less mobile, and bioavailable as compared with the radiocesium of condensation particles [25,26,27,28]. Condensation particles formed following the Chernobyl accident were similar to the global fallout after nuclear weapon tests, and their behavior in the environment could be foreseen with fairly good precision [21]. At the same time, before the Chernobyl accident, it was not known how fuel particles could behave, and, therefore, this was a major scientific challenge [5,21,23,27].

Due to the presence of water-insoluble fuel particles, the mobility and bioavailability of deposited radiocesium were low in the nearby zone and depended on the distance from the damaged unit [7,27]. For example, the fraction of nonexchangeable ^137^Cs in the fallout near Chernobyl was about 75% [21,28]; in the Bryansk region, it was 40–60% [27]; and in Cumbria (UK), it was about 10% [29]. Therefore, the Chernobyl radiocesium was less mobile and bioavailable in the area close to the NPP than in remote areas, especially in Western Europe [29,30,31,32].

Radionuclide leaching from fuel particles was the key process with respect to the transformation of its speciation in a soil–water environment after the Chernobyl accident in the midterm and for some specific conditions in the long term [5,21,27,33,34,35,36]. Radionuclides are released from fuel particles by three mechanisms: (1) due to the diffusion from the particle to the surface and passing into the solution; (2) by mechanical disintegration of the particle and the correspondent increase in the surface of the solid–liquid interface; and (3) with the dissolution of the fuel matrix [33]. Pure uranium dioxide (nuclear fuel) is known to slowly dissolve even in concentrated acids [37]. In natural conditions, however, tetravalent uranium U(IV) is easily oxidized by atmospheric oxygen to its hexavalent U(VI) state, which more readily dissolves in aqueous media compared with U(IV) [35,37]. The crystals of UO_2_, when in water, are quickly covered by an oxide film of UO_2+x_, where 0 < x < 1, and the rate of further oxidation is determined by the diffusion of oxygen and the surface area of the crystal. In addition to oxygen, UO_2_ can be oxidized by other oxidizers such as trivalent iron Fe (III) or hydrogen peroxide, molecules of which can be formed in the vicinity of the hot particle as a result of water radiolysis. The dissolution of oxidized uranium is facilitated by the presence of inorganic carbonates and sulfates, as well as organic ligands [37].

Bogatov et al. (1990) stated that the main mechanism of radionuclide leaching from fuel particles was the dissolution of the uranium matrix. In their study, the dissolution rate was estimated to be (0.57–16) × 10^−5^ g/cm^2^day at the contact of particles of different size with solutions modeling aqueous media [20]. The diffusion release rate for radionuclides was determined by these researchers using the method of layer dissolution of particles. Based on the depletion of surface layers, the diffusion coefficients were estimated to be close and equal to (2–4) × 10^−17^ cm^2^/s for ^137^Cs, ^90^Sr, ^238,239,240^Pu, ^241^Am [20]. The ability of fuel particles, being aggregates of fine crystals of uranium oxides, to easily break down at physical impact was indicated by many authors. The breakdown of fuel particles is facilitated by a large number of radiation–thermal defects in their structure. As a result of disintegration, the mean size of hot particles is decreasing, and the part of particles with high activity is declining. Consequently, the rate of the radionuclide release from the fuel matrix by both mechanisms (diffusion and dissolution) should be growing [7,27].

#### 2.1.2. Fukushima

Following the FDNPP accident, radiocesium was first assumed to have deposited in water-soluble forms [38] and transported in the atmosphere by sulfate aerosol particles of 0.5–0.6 μm diameter. Yet, spherical glassy hot particles of a few micrometers in diameter were discovered later as far as 170 km from the FDNPP, containing, apart from radiocesium, uranium and other elements representative of reactor materials [39,40]. Similar particles have been identified by Niimura et al. (2015) using autoradiography of soils, plants, and mushrooms [41]. Near the FDNPP, even coarser particles (up to hundreds of micrometers) were later identified with higher radiocesium activity (sometimes more than 1000 Bq per particle) and irregular shape [42]. These radiocesium-bearing microparticles (CsMPs) are primarily composed of SiO_2_ [43]. In terms of radiocesium fate and transport, it is important that CsMPs are insoluble in water and persistent in the environment [42,44]. For adequate modeling and prediction of Fukushima-derived radiocesium behavior in the environment, it is necessary to know the fraction of CsMPs activity in the total radiocesium release and deposition at different locations and the rate of radiocesium leaching from CsMPs due to decomposition.

Quantitative characterization of CsMPs in surface soils at different directions from the FDNPP was performed in [45,46]. The amount of CsMPs in surface soils at various directions, distances, and deposition levels in the soils was 0.9–101 particles/g, and the fraction of radiocesium embedded in CsMPs out of the total amount of deposited radiocesium was 15–80%. The presence of CsMPs in soils and sediments has a major impact on the radiocesium solid–liquid distribution in the soil–water environment [47]. The fact that the fraction of CsMPs in the deposition significantly varies in different locations makes it difficult to model the radiocesium behavior. As a result, uncertainty in the prediction of the mobility and bioavailability of radiocesium in the soil–water environment and its dynamics is increasing [48].

CsMPs were also identified in the suspended sediments of the Kuchibuto River, which is one of the most contaminated tributaries of the Abukuma River, where the fraction of radiocesium embedded in CsMPs out of the total amount of Fukushima-derived radiocesium in sediments was up to 67% [49].

### 2.2. Transformation of Basic Radiocesium Chemical Forms in Soil–Water Environment

After the soil–water environment is contaminated, the initial chemical forms of radionuclides are subject to transformation [5,7,27,50,51]. With hot particles (fuel particles in Chernobyl [5,7,27,33,34,36] and CsMPs in Fukushima [52,53,54]) disintegrated, radiocesium incorporated in these particles transfers to the solution. In the solution, radiocesium is sorbed by the soil and sediment particles by ion exchange [5,7,27,53,55,56,57,58,59,60,61,62,63]. Exchangeably sorbed radiocesium is fixed by micaceous clay minerals due to the replacement of the interlayer K-cations by Cs-cations [5,7,55,57,58,64,65]. Fixation of radionuclides is understood as the transformation of their exchangeable form to nonexchangeable. Some researchers believe that the mechanism of radiocesium fixation consists of the replacement of interlattice K^+^ by Cs^+^ ions due to the collapse of the expanded edges of the mineral’s crystal interlayers and/or the slow long-term solid-state diffusion of Cs^+^ ions through the interlayer inside the particle [27,57,58].

Initially, radiocesium fixation was treated as an irreversible process [58]. However, data about long-term transformation of radionuclide chemical forms in the soil after nuclear weapon testing [66] and Kyshtym accident [21] and 36-year studies after the Chernobyl accident are indicative of the existence of the remobilization process, which is the reverse of fixation [5,67,68]. After the deposition of radiocesium onto the soil, the fraction of its exchangeable form is not decreasing to zero, as expected for irreversible fixation, but only decreasing to a certain steady-state level and then not significantly changing [7,21]. The mechanism of radiocesium remobilization is similar to the mechanism of radiocesium leaching from hot particles: disintegration or weathering of mineral particles with a newly emerging solid–liquid interface.

**Figure 1:**
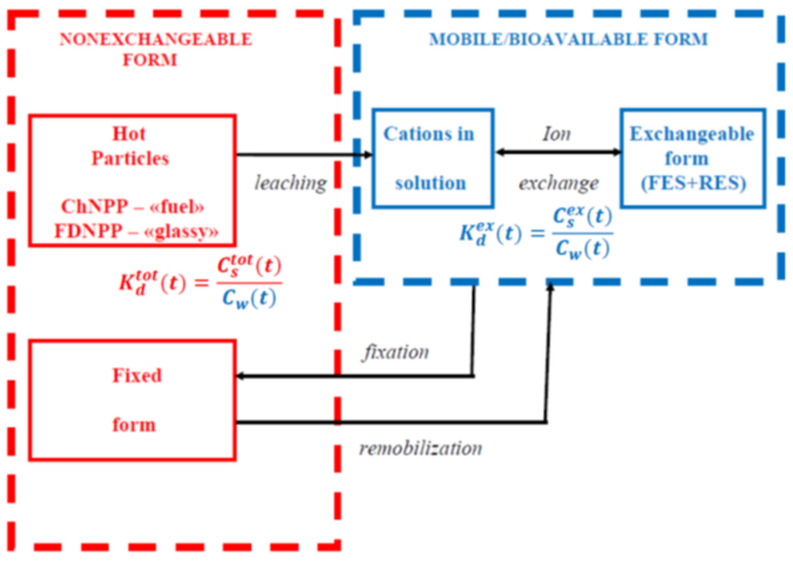
Scheme of basic transformation processes of radiocesium speciation in soil–water environment (modified after [27,53]). Blue compartment includes dissolved and exchangeable radiocesium (mobile and bioavailable forms; Red compartment includes non-exchangeable forms of radiocesium (excluded from exchange with water in immediate term). Reprinted/adapted with permission from Ref. [27]. Copyright year 2020, copyright owner’s name SPRINGER Nature.

The key transformation processes for radiocesium chemical forms (both Chernobyl and Fukushima) in the soil–water environment are shown in Figure 1.

#### 2.2.1. Solid–Liquid Distribution of Radiocesium in Soil–Water Environment in Fukushima and Chernobyl

The solid–liquid distribution of radiocesium is a governing factor for its fate and transport in the soil–water environment. It is characterized by the apparent distribution coefficient *K_d_* (L/kg), which is the ratio of the particulate radionuclide activity concentration [*R*]*_p_* (Bq/kg) to its dissolved activity concentration [*R*]*_d_* (Bq/L) [69]:

(1) $$K_{d}=\frac{{\left[R\right]}_{p}}{{\left[R\right]}_{d}}$$

[*R*]*_p_* includes the radiocesium embedded in hot particles (fuel particles in Chernobyl and CsMPs in Fukushima), the exchangeably sorbed radiocesium, and radiocesium fixed by clay minerals in sediments (Figure 1). The exchangeable radiocesium occurs at an instantaneous ion-exchange equilibrium with the liquid phase, whereas the nonexchangeable form does not take part in the radiocesium exchange with the solution. Therefore, it is worth using the exchangeable distribution coefficient $K_{d}^{ex}$, which is the ratio of the exchangeable radionuclide activity concentration in sediments [*R*]*_ex_* to its activity concentration in the solution at equilibrium [*R*]*_d_* [5,7,70,71]:

(2) $$K_{d}^{ex}=\frac{{\left[R\right]}_{ex}}{{\left[R\right]}_{d}}=\alpha_{ex}K_{d}$$

where $\alpha_{ex}$ is the exchangeable fraction of radiocesium in the sediments, and $K_{d}$ is the apparent (or total) distribution coefficient.

The exchangeable sorption of radiocesium onto the soil and sediment particles can be selective or nonselective depending on the type of sorption sites [7,27,55,56,72]. The diversity of sorption sites can be divided into two main types: regular exchange sites (RESs) and selective sorption sites occurring on the frayed edges of the neighboring layers of micaceous clay crystal lattice (FES). The selectivity coefficient of regular radiocesium sorption on the RES non-selectively, i.e., of its sorption in relation to K^+^, NH_4_^+^, and other monovalent cations, is close to one. On the other hand, the selectivity coefficient for Cs sorption on the FES is approximately 1000 for K^+^ and approximately 200 for NH_4_^+^ [73]. The FES constitutes a relatively small portion of cation exchange capacity (1% to 5%) for most soils and sediments [56]. Since FESs are characterized by high selectivity for cesium and because radiocesium (and even stable cesium) occurs at trace concentrations in the environment, the exchangeable radiocesium becomes concentrated on the FES in most sediments and soils.

The exchangeable distribution coefficient can be estimated using the standard geochemical characteristics of soils and sediments such as the capacity of sorption sites and water cation composition [27,53,70,71,74,75,76,77]. Conversely, the value of the apparent *K_d_* cannot be predicted only on the basis of environmental characteristics.

The ability of the sediment to selectively sorb radiocesium can be described using the capacity of selective sorption sites ([FES]) or radiocesium interception potential (RIP), with the RIP being a product of the [FES] and cesium selectivity coefficient with respect to a competitive ion.

A method is available for the quantitative determination of the FES capacity [FES] and RIP [56,78], and this method was modified with the consideration of exchangeable distribution coefficient $K_{d}^{ex}$ [74,75]. In this context, the exchangeable radiocesium interception potential $RIP^{ex}\left(K\right)$ is defined as follows:

(3) $$RIP^{ex}\left(K\right)=K_{c}\left(Cs/K\right)\left[FES\right]=K_{d}^{ex}\left[K^{+}\right]$$

where *K_c_*(*Cs*/*K*) is the selectivity coefficient for the ion exchange of Cs^+^ on the FES with respect to cation K^+^. $RIP^{ex}\left(K\right)$ is the intrinsic property of a given sediment accounting for its ability to selectively and reversibly sorb cesium. Given that both potassium and ammonium are relevant for radiocesium desorption, the expression can be written as follows [27,73,74]:

(4) $$RIP^{ex}\left(K\right)=K_{d}^{ex}\left(\left[K^{+}\right]+K_{c}^{FES}\left(NH_{4}/K\right)\left[N{H_{4}}^{+}\right]\right)$$

where $K_{c}^{FES}\left(NH_{4}/K\right)$ is the selectivity coefficient of ammonium in relation to potassium for the FESs. As shown by numerous post-Chernobyl studies, most soils and sediments are characterized by $K_{c}^{FES}\left(NH_{4}/K\right)=5\pm 2$; therefore, Equation (4) can be approximated by [73,74]

(5) $$K_{d}^{ex}=\frac{RIP^{ex}(K)}{\left[K^{+}\right]+5\left[N{H_{4}}^{+}\right]}$$

**Figure 2:**
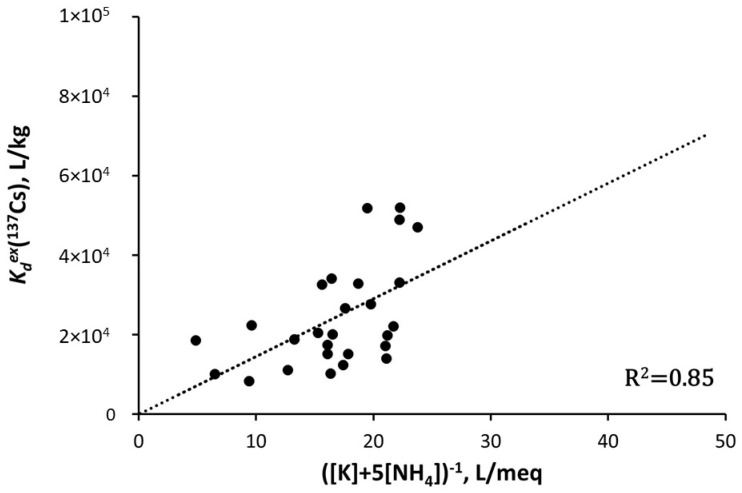
Correlation between $K_{d}^{ex}$ and inverse effective concentration of major Cs competitive cations ${\left(\left[K^{+}\right]+5\left[N{H_{4}}^{+}\right]\right)}^{-1}$ for Funasawa pond in close vicinity of FDNPP in 2015–2019 [79]. Reprinted/adapted with permission from Ref. [79]. Copyright year 2020, copyright owner’s name SPRINGER Nature.

The relationship of $K_{d}^{ex}$ (L/kg) and ([*K*^+^] + 5[*NH*_4_^+^])^−1^ (L/mmol) was shown to be linear for the ponds in the vicinity of the FDNPP, with the slopes corresponding to the *RIP^ex^*(*K*) values calculated to be about 2000 mmol/kg [53,79]; see Figure 2 for the pond Funasawa, 3.5 km to SW from the FDNPP.

Wauters et al. (1996) characterized a variety of European soils and sediments (overall, 120 samples) contaminated after the Chernobyl accident in terms of the cation exchange capacity and RIP [78]. They found that the RIP varied in a wide range from 100 to 10,000 mmol/kg depending on the soil type, cation exchange capacity, and clay content.

Contaminated soils in Fukushima, as compared with typical Chernobyl soils, show a relatively high content of clay minerals (up to 30% or more), including a sufficient amount of micaceous minerals [17,80,81]. Many studies following the FDNPP accident applied the RIP theory and methodology [56,73,75,78,82] to characterize soils and sediments in terms of their ability to adsorb and fix radiocesium [80,83,84,85,86,87,88,89,90,91,92]. However, in all of them, the RIP determination protocol did not take into account the fixation of radiocesium by clay minerals, and, therefore, the obtained values are overestimates and higher than *RIP^ex^* [53,74,75]. A total of 97 paddy soils from Fukushima Prefecture were characterized in terms of the RIP in [86]. The RIP ranged from 340 to 5360 mmol/kg, with the mean being 1670 ± 870 mmol/kg. In another study, the RIP was determined for 925 agricultural soil samples collected from Fukushima Prefecture and neighboring regions. RIP values ranged from 73 to 12,700 mmol/kg and were sometimes very different for the same soil types [92]. Overall, the RIP values for Fukushima soils are comparable with or a bit higher than those for European soils and sediments obtained by the same protocol [78].

Soon after the FDNPP accident, it was discovered that Fukushima-origin radiocesium is strongly bound to the soil and sediment particles, and its apparent distribution coefficient *K_d_* in Fukushima rivers is at least an order-of-magnitude higher than in Chernobyl rivers [8,47,93,94,95]. This fact was actually confirmed by longer-term studies [95,96,97,98,99,100,101]. Table 1 presents the summary of available data on the ^137^Cs distribution in the suspended sediment–water system of rivers in Chernobyl- and Fukushima-contaminated areas. The difference in *K_d_* for Fukushima and Chernobyl soil–water environments, in our view, is associated with two factors: (1) a relatively high fraction of micaceous clay minerals in Fukushima soils and sediments capable to selectively sorb and fix radiocesium [81,83,88,89]; and (2) occurrence of high proportion of insoluble in water and persistent in the environment glassy hot particles CsMPs in FDNPP release [8,47,52,53,54,101].

#### 2.2.2. Radiocesium Leaching from Chernobyl Fuel Particles and Fukushima Glassy Hot Particle CsMPs

The processes occurring with hot particles are difficult to model due to the diversity of their size, shapes, and chemical characteristics. Therefore, an integral parameter is often used such as the first-order rate constant *k_l_* (yr^−1^) accounting for the rate of radionuclide leaching from hot particles, i.e., radionuclide transfer from a hot particle to the solution [27,33,34]. Then, a decrease in the fraction of radionuclide embedded in particles as a function of time follows the equation [5,27,33]

(6) $$\frac{dF_{t}}{dt}=-k_{l}F_{t}$$

and hence

(7) $$F_{t}=F_{0}e^{-k_{l}t}$$

where *F_t_* and *F*_0_ are the fractions of radionuclide in hot particles and initial depositions, respectively, at the time *t* since the accident.

As demonstrated by many studies on the decomposition of Chernobyl-origin fuel particles, the rate of radionuclide leaching from them in soils varies in the range 0.05–0.5 yr^−1^ [5,7,27,33,34,36,103,104], depending on the location and soil type. Based on a large amount of statistically reliable data, the dependence of *k_l_* on the soil pH was derived for different parts of the contaminated zone [103,104]. Over several years after the accident, fuel particles primarily occurred in the upper centimeters of the soil, both close to the reactor and at distances of 250 km [105]. Most of the particles were concentrated in the 0–1 cm layer, and their proportion markedly decreased with depth. The vertical profile of radioactive particles in the soil was practically independent of the distance from the NPP and is mainly governed by the soil type. The lack of dependence of the vertical distribution of particles on their size and chemical nature suggests that the primary mechanism of radionuclide migration in the upper soil layer is its mixing by soil flora and fauna.

To date, fuel particles in the terrestrial soils of the Chernobyl exclusion zone (ChEZ) have mostly disintegrated [7,27,33]. This is not the case, however, for the bottom sediments of the cooling pond (CP) of the Chernobyl NPP and the heavily contaminated lakes of ChEZ, where the dominant fraction of radionuclides was deposited within fuel particles. Importantly, most of the radioactivity in these bottom sediments still occurs in the form of fuel particles [35,106]. In the cooling pond sediments, the dissolution of fuel particles is much slower than in soils due to low dissolved oxygen concentration and relatively high pH.

The kinetic studies of fuel particle decomposition and radionuclide leaching in the soil–water environment conducted in Chernobyl were mostly based on investigating radiostrontium speciation since this radionuclide is weakly fixed by soils and sediments [21,34,107]. Yet, the rate constants of ^137^Cs and ^90^Sr leaching from fuel particles appear to be similar [5,21,26], as shown by the kinetic calculations based on the conceptual model (see Figure 1). For most typical soil types in the Chernobyl 30 km zone, the rate constant of ^137^Cs leaching from fuel particles was in the range 0.1–0.5 yr^−1^.

In the case of Fukushima, radiocesium leaching from CsMPs can be expected to be comparable or even slower than that from Chernobyl-origin fuel particles, and when released from CsMPs, radiocesium in the soil–water environment is fairly quickly fixed by the clay minerals of soils or sediments. In laboratory experiments with individual CsMPs, Okumura et al. (2019) showed that radiocesium can be leached by weathering in the environment with a rate dependent on the temperature and water composition [54]. Based on the data of this study, we attempted to determine the rate constants *k_l_* for radiocesium leaching from CsMPs. At 30 °C, the average value of *k_l_* for three individual CsMPs was obtained to be 0.14 ± 0.01 yr^−1^ in pure (deionized) water and 2.1 ± 0.4 yr^−1^ in seawater. The temperature dependence of the CsMP dissolution in the temperature range 30–120 °C was characterized by the activation energy of 65 kJ/mol for pure water and 88 kJ/mol for seawater [54]. Extrapolating the derived temperature dependence to the mean annual temperature in Fukushima of about 13 °C, we arrived at *k_l_* ≥ 0.043 year^−1^ in freshwater and *k_l_* = 0.44 year^−1^ in seawater. It should be said that our estimate for freshwater is the lower-bound value. From the standpoint of composition, freshwater is intermediate between pure water and seawater, and, therefore, the rate constant for freshwater can be expected to be higher than for pure water but lower than for seawater.

The rate constant can also be estimated from indirectly monitoring data. Time dependence of particulate and dissolved ^137^ Cs concentrations derived from monitoring data and ^137^ Cs apparent distribution coefficient *K_d_* can then be used to determine k_l_. The reasoning behind this approach was as follows. Monitoring of ^137^Cs in three heavily contaminated ponds in the vicinity of the FDNPP revealed a gradual decline of its apparent solid–liquid distribution coefficient *K_d_* from 2015 to 2019 [52,53], which is not typical of radiocesium dynamics in water bodies in the mid- and long term [18,102]. The initial and intermediate phases after the Chernobyl accident, and after nuclear weapon tests (NWTs), were characterized by a slight increase in radiocesium apparent *K_d_* in soils and sediments as a result of fixation or aging [5,7,60,62]. Since leaching of ^137^Cs from CsMPs is the slowest process (limiting stage), as shown by Figure 1, it seems reasonable to assume that the *K_d_* decline trend is associated with gradual remobilization of ^137^Cs due to leaching from CsMPs. Against this background, calculations of *k_l_* were performed for three ponds, and the obtained values were in the range 0.12–0.18 yr^−1^. A similar trend for radiocesium apparent *K_d_* in 2012–2020 with the rate constant *k_l_* = 0.05 yr^−1^ has been recently reported for the suspended sediment–water system in the Abukuma River at Fukushima City (Kuroiwa) [99]. Thus, our estimates based on long-term monitoring data are consistent with those derived from laboratory experiments with individual CsMPs. It is important to note that *k_l_* for Fukushima-derived CsMPs corresponds to the lower bound of *k_l_* for Chernobyl-derived fuel particles, and, by and large, leaching of radiocesium from CsMPs is slower than from fuel particles.

#### 2.2.3. Radiocesium Fixation by Soils and Sediments and Remobilization

Fixation of radionuclides is the transformation of their exchangeable form to nonexchangeable. It is believed that the mechanism of radiocesium fixation is the replacement of interlattice K^+^ by Cs^+^ ions due to the collapse of the expanded edges of the mineral’s crystal interlayers and/or the slow long-term solid-state diffusion of Cs^+^ ions along the interlayer inside the particle [27,58,59].

Initially, radiocesium fixation was treated as an irreversible process [58]. However, data about the long-term transformation of radionuclide chemical forms in the soil after nuclear weapon testing [66] and Kyshtym accident [21] and 36-year studies after the Chernobyl accident are indicative of the existence of the remobilization process that is the reverse of fixation [5,67,68]. After the deposition of radiocesium onto the soil, the fraction of its exchangeable form is not decreasing to zero, as expected for irreversible fixation, but only decreasing to a certain steady-state level and then not significantly changing [7,21]. The mechanism of radiocesium remobilization is similar to the mechanism of radiocesium leaching from hot particles-disintegration or weathering of mineral particles with a newly emerging particle–solution interface.

As it appears from laboratory experiments with a variety of soils and sediments [59,64,77] and post-Chernobyl field studies [5,27], the timescale of radiocesium fixation equals to weeks or months (*k_f_* = 4–20 yr^−1^), depending on environmental conditions, whereas the timescale of remobilization can be up to a few years (*k_r_* = 0.4–2 yr^−1^). These processes can be expected to show comparable rates in Fukushima soils and sediments.

The first-order kinetics, however, cannot be considered as an absolutely accurate description of radiocesium fixation since this process is diffusional in character and slows down with time [77]. According to the diffusional model, radiocesium fixation is attributable to its diffusion into the surface layers of clay particles. Given that the thickness of the diffusion layer is much smaller than the particle size, fixation can be considered as the diffusion of radiocesium into a sheet. In this case, the time dependence of mobile or exchangeable fraction *M*(*t*) can be approximated by the equation [108,109,110,111]

(8) $$M\left(t\right)=M_{\infty }\left(1+\frac{\delta }{\sqrt{t}}\right)$$

where $M_{\infty }$ is the mobile fraction at equilibrium, and *δ* is the diffusional kinetic parameter equal $\frac{l}{\sqrt{\pi D}}$; *l* is the thickness of the diffusional layer, *D* is the diffusion coefficient of ^137^Cs in the solid phase of clay mineral, and *t* is time.

Importantly, only two parameters $M_{\infty }$ and *δ* are needed to describe the long-term kinetics of radiocesium fixation by soils and sediments. Unlike the first-order kinetics, the diffusional model of fixation predicts the decline of the radiocesium mobile fraction not up to zero but up to equilibrium state $M_{\infty }$. Various soil types have been characterized in terms of parameters $M_{\infty }$ and *δ* [68,111], allowing long-term predictions (Table 2).

## 3. Radiocesium Downward Migration in Soil

As time goes on, radionuclides deposited on the ground surface tend to migrate down through the soil profile. The dynamic pattern of the vertical distribution of radionuclides in the soil is critical from the standpoint of the external dose rate, availability of radionuclides for transfer to surface runoff and wind resuspension in the boundary atmospheric layer, availability of radionuclides for root uptake by plants, and penetration to groundwater [8,112,113,114,115,116]. Assessment of the radionuclide transfer from land to surface waters requires knowing the radionuclide concentration in the topsoil layer [18,116]. Radionuclides vertically migrate in the solution with infiltration water flow or attached to fine soil particles [5,112,113,117]. The transport of radiocesium in the solution by infiltration is slower than the water flow because of sorption–desorption and fixation on soil particles (Figure 1). Fine soil particles containing immobile radiocesium can move by penetrating through pores, cracks, and cavities, and as a result of the vital activity of plants and biota (bioturbation) [113,118]. Nevertheless, the vertical migration of radionuclides in undisturbed soils can be described by the advection–dispersion equation using the effective values of the dispersion coefficient and advective velocity [5,112,116,117,119,120]. The most accurate way of representing radiocesium migration by the advection–dispersion model is the simultaneous solution of respective equations separately written for specific radiocesium chemical forms in soil *R_i_* with allowance for their transformation [5,121]:

(9) $$\frac{\partial R_{i}}{\partial t}=\frac{\partial }{\partial x}\left(D_{i}\frac{\partial R_{i}}{\partial x}\right)-v_{i}\frac{\partial R_{i}}{\partial x}+\sum k_{ji}R_{j}-\sum k_{ij}R_{i}$$

with the initial conditions:

$$R_{i}=R_{i}^{0}\delta \left(x-0\right)$$

and boundary conditions:

$$R_{i}{|}_{x=\infty }=0$$

where *D_i_* and *v_i_* are the effective dispersion coefficient and effective advective velocity for each chemical form *i,* respectively, and *k_ij_* is the rate constant of transformation of chemical form *i* to *j*.

In radioecological studies, for one-time release, the model is often used in its simplified version based on the approximation of the analytical solution of Equation (9) for the total radionuclide concentration *R* = ∑*R_i_* [112,116,119,120,122]:

(10) $$R\left(x,t\right)=\frac{\sigma_{0}}{\sqrt{\pi D_{eff}t}}e^{-\left(\frac{{\left(x-vt\right)}^{2}}{4D_{eff}t}+\lambda t\right)}$$

where *σ*_0_ is the initial radionuclide deposition on the soil, and λ is the rate constant of radionuclide decay. This approximation is valid for long term: *t >>* 2*D*/*v*^2^ [122]. The studies of the vertical migration of radiocesium in undisturbed soils of grassland and forests showed that, as a rule, ^137^Cs transport due to dispersion prevails over advective transport [18,102,112,113,115,116]. Therefore, Equation (10) can be further simplified:

(11) $$R\left(x,t\right)=\frac{\sigma_{0}}{\sqrt{\pi D_{eff}t}}e^{-\left(\frac{x^{2}}{4D_{eff}t}+\lambda t\right)}$$

Radiocesium in the Fukushima soils was migrating faster than in the Chernobyl zone, as was revealed soon after the FDNPP accident [8,114,123,124] for the top layer of the contaminated soil. There can be several factors responsible for this fact. Firstly, the mean annual precipitation in the Fukushima area is more than two times higher than that in the Chernobyl area [8,79]. Hence, a more active infiltration flow can lead to higher migration rates of both mobile and immobile forms of radiocesium, which are entrained by infiltration flow when moving down the soil through pores and cracks [113,114].

**Figure 3:**
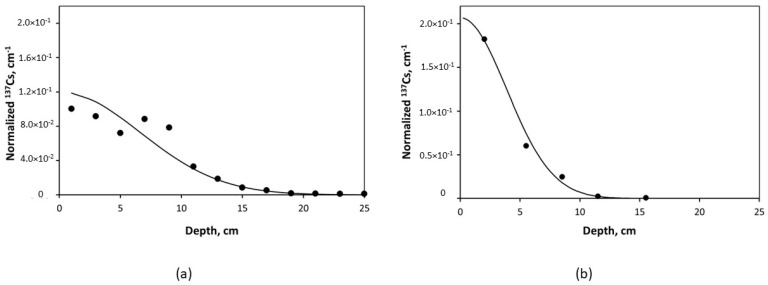
^137^Cs differential distribution in undisturbed alluvial meadow soils -measurements (points) against diffusional model Equation (11) (line). (**a**) soil core collected in ChNPP exclusion zone near village Benevka (9 km NW from ChNPP) in 2017; model fit with *D_eff_* = 0.7 cm^2^/yr [102] Reprinted/adapted with permission from Ref. [118]. Copyright year 2016, copyright owner’s name SPRINGER Nature. (**b**) soil core collected in FDNPP exclusion zone at Okuma town on the catchment of Suzuuchi pond (3.75 km NW from FDNPP); model fit with *D_eff_* = 2.5 cm^2^/yr [114]. Levels of ^137^Cs soil contamination are presented as volumetric activity concentration normalized by ^137^Cs deposition (1 cm^−1^ = Bq∙cm^−3^/Bq∙cm^−2^). Reprinted/adapted with permission from Ref. [118]. Copyright year 2016, copyright owner’s name SPRINGER Nature.

Figure 3 presents a comparison of the ^137^Cs vertical distribution in the undisturbed alluvial meadow sandy soil of ChEZ at Benevka in 2017 (31 years after the ChNPP accident) [102] with that in the FDNPP exclusion zone at Okuma town (catchment of Suzuuchi pond) in 2014 (3 years after the FDNPP accident) [114].

**Figure 4:**
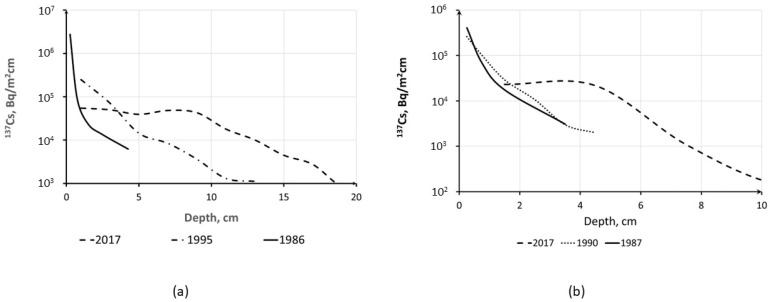
Long-term time changes in ^137^Cs vertical distribution in soils of ChNPP exclusion zone nearby villages Benevka (**a**) for 1986–2017 and Korogod (**b**) for 1987–2017 [102]. Reprinted/adapted with permission from Ref. [102]. Copyright year 2020, copyright owner’s name SPRINGER Nature.

The long-term dynamics of the ^137^Cs vertical distribution in the soil of the ChEZ is illustrated in Figure 4. It should be noted that the maximum ^137^Cs activity concentration is still located in the topsoil layer, and profiles can be approximated by Equation (11). It is interesting to note that a recent study [27] showed an s profile at Benevka for 2017 in comparison with the profile of ^241^Am. Activity concentrations of ^137^Cs and ^241^Am differ by more than an order of magnitude, yet the shape of the profiles is similar. Since the physicochemical properties of ^137^Cs and ^241^Am including their soil–water distribution are different, similarities of their profiles indicate that they move downward in the soil with particles in which both are incorporated, and their migration in the solution with infiltration flow does not play a significant role [102]. It can be expected that this is even truer for Fukushima-derived radiocesium since it is strongly bound by soil particles than Chernobyl-derived radiocesium.

## 4. Time Changes of Radiocesium Concentrations in Freshwaters

In case of a nuclear accident, radioactive contamination of water reservoirs and rivers running through the affected areas is a major challenge since these water bodies are a source of drinking water for the public and used for fishing and irrigation. As a result of the Chernobyl accident in 1986, extensive areas of the Dnieper River basin, including the watershed of its right tributary, the Pripyat River, were contaminated with ^137^Cs [5,6,9,18,124,125,126]. Radionuclides then go beyond the initially contaminated areas and across boundaries due to transport by the river systems [123]. After the FDNPP accident in 2011, the river basins of the Abukuma, Mano, Niida, Ohta, Ukedo, Maeda, Kuma, and others [13,93,94,95,96,97,98,99,100,101,127,128] flowing into the Pacific Ocean were also exposed to contamination, with ^137^Cs transported both in a particulate and dissolved state.

Initial radioactive contamination of water bodies after the nuclear accidents at the ChNPP [3,6,124,125] and FDNPP [93,94,95,96,97,98,99,100,101,127,128] was relatively high, owing to the direct fallout onto the river and lake surfaces. The contamination of water bodies was then sharply decreasing due to the fast processes of sorption and fixation of radionuclides to sediments, as well as the sedimentation of particles to the bottom [6,124]. Yet, extensive territories contaminated due to the accidents continue to serve as a long-term source of radioactivity to natural waters and aquatic ecosystems. Wash-off driven by surface runoff is the primary pathway for the contamination of water bodies in the mid- and long term [5,6,9,11,96,99,100,101,102,129,130,131,132,133,134,135,136,137,138].

### 4.1. Long-Term Dynamics of Radiocesium in Rivers and Lakes and Its Prediction

Modeling and prediction of radionuclide long-term behavior in the environment are keys for the management of contaminated areas. In post-Chernobyl studies, temporal changes in dissolved ^137^Cs concentrations in rivers were often described by the empirical fitting model using a series of exponential functions [6,102,133,134,135]:

(12) $$c\left(t\right)=\sum_{i}c_{i}^{0}e^{-\left(\lambda +k_{i}\right)t}$$

where *c*(*t*) is the current ^137^Cs activity concentration in the river (for particulate ^137^Cs in Bq/kg, and for dissolved in Bq/m^3^); *λ* is the ^137^Cs decay rate constant equal 0.023 yr^−1^; *k_i_* are the empirically fitted rate constants; $c_{i}^{0}$ are the fitting parameters representing the initial concentration of the ith individual exponential function (in the case of a three-exponential model, *i* can take a value of 1, 2, and 3), and *t* is the time. The same approach was followed by a number of researchers in post-Fukushima studies of radiocesium (both dissolved and particulate) dynamics in rivers [92,93,94,95].

In the case of Chernobyl, the contamination analysis and prediction were focused on dissolved ^137^Cs since it was predominant in the contaminated natural waters and controlling radionuclide transport [18,126,136,137,138]. As to the particulate concentration of ^137^Cs in surface waters, such data were scarce. There are only two cross-sections in the Chernobyl-contaminated area—for the Pripyat River at Chernobyl and for the Dneper River at Nedanchichi, for which long-term monitoring data are available [18,102]. Time dependence of annual mean particulate ^137^Cs activity concentrations for these two cross-sections was approximated by a two-exponential model (Equation (12)) using the fitting parameters presented in Table 3.

The above empirical model (Equation (12)) based on the multiexponential description of radionuclide dynamics, however, does not seem to adequately reflect the actual mechanisms underlying the changes of radionuclide activity concentrations in water. Unfortunately, this approach requires using a number of functions accounting for short-, middle-, and long-term phases after the accident, as well as parameters that are not initially known.

An alternative way to model mid- and long-term dynamics of radiocesium in rivers is a semiempirical diffusional approach [18,102]. The key assumption of the model is that the main source of suspended particles for surface runoff is the top layer of catchment soil, and radiocesium concentration in the topsoil layer is described by the simplified Equation (11). In this case, the radiocesium concentration in the topsoil layer and thus in suspended sediments can be approximated by the following equation:

(13) $$C_{p}\left(t\right)=\frac{\sigma_{0}}{\rho \sqrt{\pi D_{eff}t}}e^{-\lambda t}=C_{p}^{0}\;\frac{e^{-\lambda t}}{\sqrt{t}}$$

where *σ*_0_ is the initial average deposition of radiocesium on the catchment; *D_eff_* is the effective dispersion coefficient, averaged over the catchment area; λ is the radioactive decay rate constant; *ρ* is the average bulk density of the topsoil over the catchment; and *t* is the time.

The advantage of this approach is that the same equation can be used for middle- and long-term phases after a nuclear accident with the same values of physically based parameters, which can be estimated or determined by field or laboratory studies. More simply, decay corrected particulate r-Cs activity concentrations in surface runoff and rivers are described by the inverse square root of time function.

**Figure 5:**
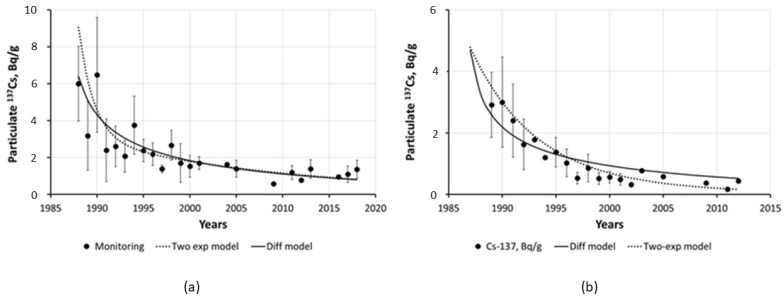
Time dependence of annual mean particulate ^137^Cs activity concentrations (black circles) in 1989–2018 for Pripyat River at Chernobyl (**a**) and in 1989–2012 for Dneper River at Nedanchichi (**b**) against approximations by Equation (13) (solid line) and by two-exponential Equation (12) with parameters from Table 3 (dotted line) [102]. Reprinted/adapted with permission from Ref. [102]. Copyright year 2020, copyright owner’s name SPRINGER Nature.

Figure 5 presents temporal changes in particulate ^137^Cs in river water over 30 years for two large rivers of the Chernobyl area: Pripyat and Dneper [105]. The significant scatter in the experimental data on ^137^Cs activity concentrations in river water, particularly in the first years after the accident, can be attributed to the extremely non-uniform distribution of radionuclides on the catchment and the occurrence of hot fuel particles, as well as uncertainties associated with sampling, processing, and measurements.

Both the semiempirical diffusional and empirical two-exponential models can be seen to account equally well for the overall trend in the long-term dynamics of particulate ^137^Cs in the rivers of the Chernobyl-contaminated areas. With the diffusional model, such long-term description becomes possible with only a single parameter $C_{p}^{0}=\frac{\sigma }{\rho \sqrt{\pi D_{eff}}}$, which is determined based on using physically meaningful and understandable characteristics such as average deposition on catchment and the effective coefficient of radionuclide dispersion in catchment soils *D_eff_*. Values of *D_eff_* in different soils are readily available in the literature [5,113,114,119,121,139,140]. Moreover, $C_{p}^{0}$ can be estimated from monitoring data of radionuclide concentration on suspended matter in rivers, given that it is no longer the initial phase. With this in mind, long-term prediction can be undertaken using the diffusional model (see Equation (13)). Meanwhile, the two-exponential model requires four fitting parameters, which are not possible to immediately determine after the accident, necessitating long-term field observations for their estimation.

With allowance for Equations (1) and (13), the time dependence of the dissolved ^137^Cs concentration in a river can be approximated by the equation [18,102]

(14) $$c_{d}\left(t\right)=\frac{\sigma_{0}}{{\rho K}_{d}\sqrt{{\pi D}_{eff}t}}e^{-\lambda t}$$

For the mid- and long-term phases after the Chernobyl accident, ^137^Cs concentrations in rivers were successfully described based on the semiempirical diffusional model (Equations (13) and (14)) using only two key physicochemical parameters: radiocesium dispersion and distribution coefficients (*D_eff_* and *K_d_*).

**Figure 6:**
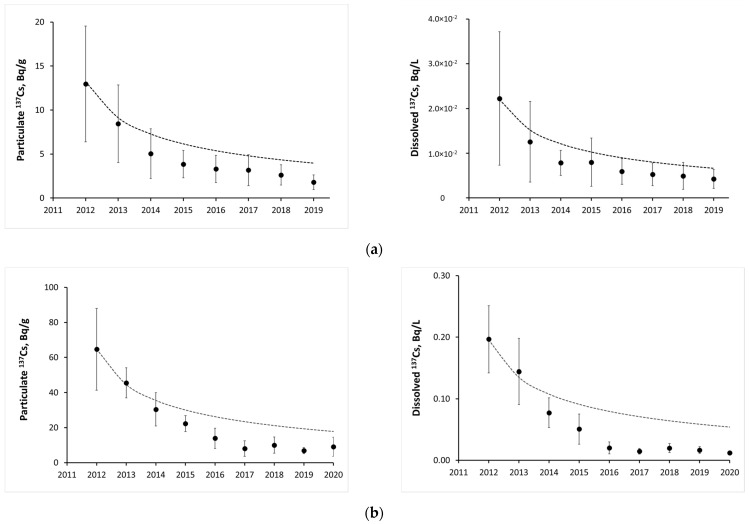
Time changes of particulate and dissolved ^137^Cs in two rivers of Fukushima-contaminated area: (**a**) annual mean particulate dissolved ^137^Cs activity concentrations in Abukuma River at Kuroiwa (Fukushima city) in 2012–2019 based on the data of [99] against diffusional model prediction (dotted line); (**b**) annual mean particulate and dissolved ^137^Cs activity concentrations in Hiso River in 2012–2020 based on the data of [100] against diffusional model prediction (dotted line).

For the Fukushima-contaminated areas, the ^137^Cs activity concentrations in rivers and lakes decline a bit faster than predicted by the semiempirical diffusional model. This is illustrated in Figure 6 showing the dynamics of the particulate and dissolved ^137^Cs in two Fukushima rivers Abukuma [99] and Hiso [100] in comparison with the predictions by the diffusional model.

There are two major potential reasons for the observed discrepancy and difference from the situation in Chernobyl. First, an extensive remediation program implemented by the Japanese government on the contaminated catchments after the accident has effectively reduced the average ^137^Cs deposition on the catchment soils and especially decreased the ^137^Cs content in the topsoil layer. Second, the basic processes of surface runoff development in Fukushima markedly differ from those in Chernobyl area due to higher intensity of precipitation, especially during typhoons, and higher energy of surface runoff flows. As a result, deeper soil layers become involved in exchange with surface runoff, which causes a faster decline of particulates and dissolved radionuclide concentration in the surface runoff.

### 4.2. Radiocesium Wash-Off from Contaminated Watersheds and Its Dynamics after the Accident

There are two parameters used to characterize the catchment-to-river transfer of radionuclides by surface runoff: the particulate and dissolved wash-off ratios *N_p_* and *N_d_* defined as [5,18,101,131,132]

(15) $$N_{p}=\frac{\bar{c_{p}}}{\sigma };\;N_{d}=\frac{\bar{c_{d}}}{\sigma }$$

where $\bar{c_{p}}$ and $\bar{c_{d}}$ are the particulate and dissolved radionuclide annual mean activity concentrations (Bq/m^3^), respectively; *σ* is the current average radionuclide inventory on the catchment (Bq/m^2^).

The particulate wash-off ratio accounts for the proportion of radionuclide inventory washed off on suspended matter by surface runoff causing the topsoil erosion of 1 kg/m^2^. The physical meaning of the dissolved wash-off ratio is that it is the proportion of radionuclide inventory washed off in the solution by a surface runoff of 1 m depth [5,18]. The wash-off ratios enable predicting the radionuclide wash-off from the contaminated catchment and its concentration in rivers and other water bodies [141,142,143,144]. To estimate the fraction of the radionuclide washed off in the solution, the dissolved wash-off ratio is multiplied by the expected runoff depth for a given runoff event or period of interest. The fraction of radionuclide washed off with sediments is estimated by multiplying the particulate wash-off ratio by the predicted sediment yield during the runoff event or period of interest [5,18,101,136]. This approach was used to predict the secondary contamination of water bodies due to snowmelt or rainfall floods after the Chernobyl accident in the early phase [5,137,138].

**Figure 7:**
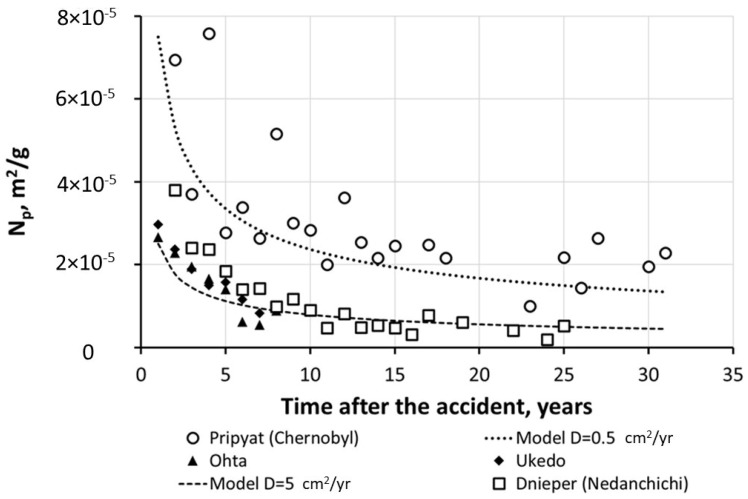
Comparison of time variations in mean annual values of particulate ^137^Cs wash-off ratios N_p_ for catchments of Pripyat River at Chernobyl and Dnieper River at Nedanchichi in Chernobyl area (based on data [102]), and for the rivers Ukedo and Ohta in Fukushima area (based on data from [95,97,98]), and comparing with semiempirical diffusional modeling at *D_eff_* = 0.5 cm^2^/year and *D_eff_* = 5 cm^2^/year [18,79,144]. Reprinted/adapted with permission from Ref. [79]. Copyright year 2022, copyright owner’s name SPRINGER Nature.

The values of *N_p_* are similar for Fukushima and Chernobyl areas when compared at similar times post-accident [18,101]. The same is true for *N_p_* obtained in runoff plot experiments in Fukushima [141,142] with data from Chernobyl [5,101,130,132,136,143] for an early time after the accident. This is illustrated in Figure 7 [18] comparing the magnitudes and time variations of the mean annual values of particulate ^137^Cs wash-off ratios *N_p_* for the catchments of the Pripyat River at Chernobyl and the Dnieper River at Nedanchichi in the Chernobyl area, and for the rivers Ukedo and Ohta in the Fukushima area and comparing with the semiempirical diffusional modeling at *D_eff_* = 0.5 cm^2^/year and *D_eff_* = 5 cm^2^/year.

**Figure 8:**
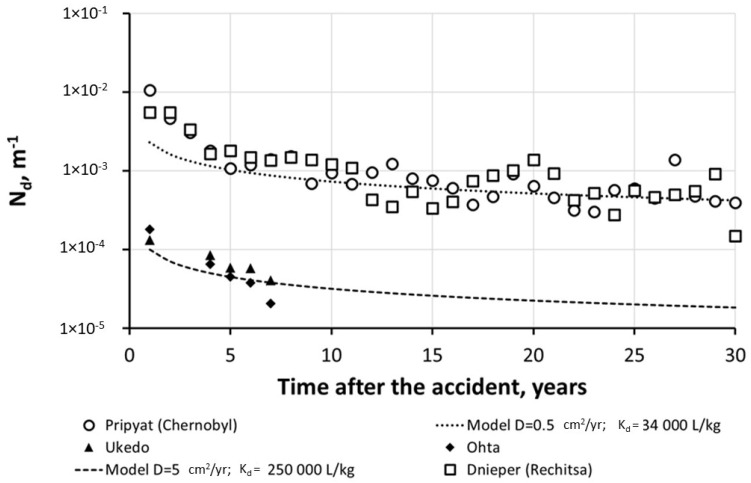
Comparison of mean annual dissolved ^137^Cs wash-off ratios *N_d_* from catchments of Ukedo and Ohta Rivers in Fukushima area (based on data from [97,98]) with those for Pripyat River at Chernobyl and Dnieper River at Rechitsa in Chernobyl area (based on data from [102]) and with estimation by semiempirical diffusional model [18,79,144]. Reprinted/adapted with permission from Ref. [79]. Copyright year 2022, copyright owner’s name SPRINGER Nature.

Figure 8 shows a comparison of the magnitudes and their time changes of the mean annual dissolved ^137^Cs wash-off ratios *N_d_* (m^−1^) for two river catchments (Ukedo and Ohta) in the Fukushima area with two river catchments Pripyat (at Chernobyl) and Dnieper (at Rechitsa) in the Chernobyl [18,79,144].

As can be seen in Figure 8, the *N_d_*(^137^Cs) values for two cross-sections, Chernobyl (Pripyat) and Rechitsa (Dnieper), are similar, and their change over time is well-described by the semiempirical diffusional model in Equation (9), provided *D_eff_*(^137^Cs) = 0.5 cm^2^/year [114,116,119] and *K_d_*(^137^Cs) = 3.4 × 10^4^ L/kg [18,102]. For the catchments of the rivers Ukedo and Ohta in the Fukushima area, the *N_d_*(^137^Cs) values are an order-of-magnitude lower than those for Chernobyl. This difference is mainly explained by the values of *K_d_*(^137^Cs), which are at least an order-of-magnitude higher for most of the rivers in the Fukushima area [47]. The changes in *N_d_*(^137^Cs) values over time for both catchments of the Ukedo and Ohta rivers are accounted by the proposed diffusional model for dissolved ^137^Cs wash-off using a catchment area average of *D_eff_*(^137^Cs) = 5 cm^2^/year [114] and mean *K_d_*(^137^Cs) = 2.5 × 10^5^ L/kg [47,97,99]. The semiempirical model describes reasonably well the mid- and long-term dynamics of the particulate and dissolved ^137^Cs wash-off, both for Chernobyl and Fukushima rivers.

### 4.3. Seasonal Variation and Temperature Dependence of Radiocesium in Freshwaters

**Figure 9:**
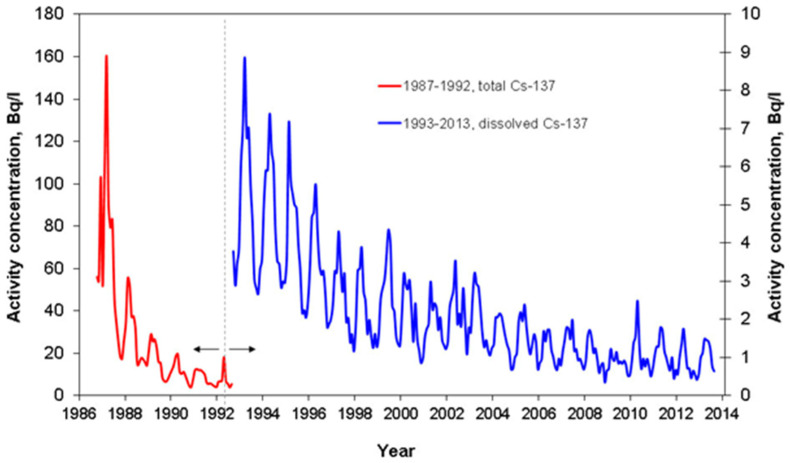
Regular seasonal variations of dissolved ^137^Cs in Chernobyl cooling pond in 1986–2014 [106]. Reprinted/adapted with permission from Ref. [106]. Copyright year 2020, copyright owner’s name SPRINGER Nature.

Based on long-term observations after the Chernobyl accident, regular seasonal variations in dissolved ^137^Cs activity concentrations were detected in the cooling pond at the ChEZ (Figure 9) [106], showing a pronounced minimum in the winter and a maximum in the summer. Similar seasonal variations were observed in the small oligotrophic lake Vorsee in Germany [74,76].

**Figure 10:**
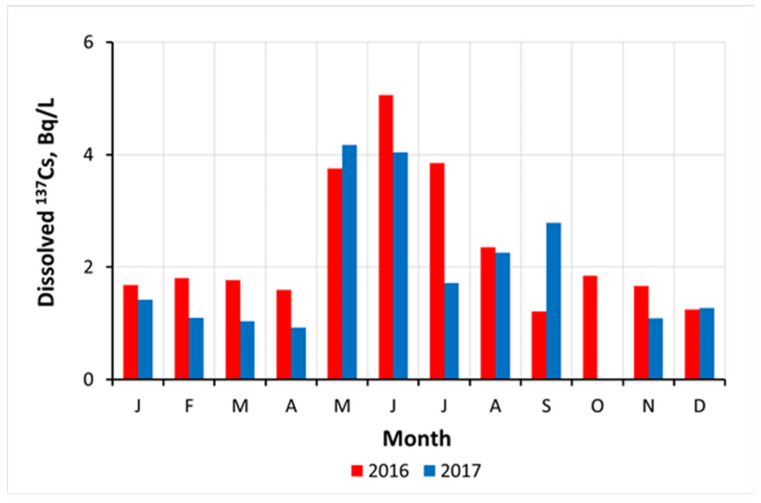
Seasonal variations of dissolved ^137^Cs activity concentration in water of Suzuuchi irrigation pond in Okuma town at FDNPP exclusion zone in 2016 and 2017 [53].

In the irrigation ponds in the vicinity of the FDNPP [53], and in the rivers of the Fukushima-contaminated areas, seasonal variations of dissolved ^137^Cs were of the same kind [97,99,145,146]. Figure 10 shows seasonal variations of dissolved ^137^Cs in water of the irrigation pond Suzuuchi located in Okuma town in 2016 and 2017. Conceivably, the seasonal changes in the water temperature could have an impact on the ion-exchange desorption of ^137^Cs, which proceeds according to the Gibbs–Helmholtz and/or Arrhenius equations [147,148]. The obtained estimates were around 20 kJ/mol for the irrigation ponds and rivers in the Fukushima-contaminated areas [53,101,146], which is consistent with the results of laboratory experiments determining E_a_ for selective sorption sites of micaceous clay minerals [148].

Seasonal variations of dissolved ^137^Cs activity concentration in the Chernobyl cooling pond [106] and lake Vorsee [74,75] were attributed to the variations in the ammonium concentration in the pond’s bottom-sediment pore water. In the Fukushima irrigation ponds, the decomposition of organic matter in the reducing conditions of bottom sediments could also have led to ammonium generation in pore water, which could have contributed, to some extent, to increased dissolved ^137^Cs in the pond’s water column in the summer [53].

## 5. Conclusions

The conducted studies showed that the processes and mechanisms underlying the environmental behavior of radiocesium in Fukushima and Chernobyl are similar. At the same time, the differences in geoclimatic and geomorphological conditions and speciation result in the differences in the quantitative parameters of radionuclide fate and transport.

Fukushima-derived radiocesium is strongly bound by the soil and sediment particles. Radiocesium apparent distribution coefficient *K_d_* in Fukushima rivers is considerably (at least an order of magnitude) higher than that in the rivers of the Chernobyl area, which is most likely due to two reasons: high binding ability of soils and sediments in the Fukushima-contaminated areas and the presence of water-insoluble hot glassy microparticles in the Fukushima accidental fallout.

Observations in the Fukushima-contaminated areas, similar to Chernobyl, have shown that the concentrations of radiocesium are higher in small lakes and ponds than in rivers and dam reservoirs. Studies of the ^137^Cs behavior in freshwater both in Chernobyl and Fukushima demonstrated regular seasonal variations: higher levels of the dissolved ^137^Cs were observed in the summer and lower levels in the winter. Additional dissolution of ^137^Cs in the summer can be attributed to the temperature dependence of ^137^Cs desorption and its remobilization by ammonium in closed and semiclosed lakes, ponds, or reservoirs.

^137^Cs activity concentrations in freshwater decreased with time after both accidents. In the case of Chernobyl, this decline in ^137^Cs concentrations is well-predicted by a semiempirical “diffusional” model. The observed ^137^Cs concentrations in Fukushima, however, declined faster than predicted by the “diffusional” model. Two potential explanations of this difference in the behavior of Chernobyl- and Fukushima-derived radiocesium were suggested: (1) extensive remediation activity during the first several years after the Fukushima accident, which substantially reduced the ^137^Cs content in the topsoil layer of the contaminated catchments; and (2) difference in surface runoff formation processes in Fukushima and Chernobyl due to higher intensity of precipitation and slopes in the Fukushima area.

Opposite to Chernobyl, the apparent distribution coefficient *K_d_*(^137^Cs) in the sediment–water system of Fukushima rivers and ponds was found to decrease with time after the accident. Proceeding on the assumption that the decrease in *K_d_* is associated with the decomposition of glassy Cs-rich microparticles, the timescale of ^137^Cs leaching from them was estimated to be in the range 6–20 years. The obtained estimates are consistent with the findings of recent laboratory experiments.

Higher mean annual precipitation and air temperature promote faster vertical and lateral radiocesium migration in Fukushima as compared with Chernobyl. Wash-off is the principal long-term process responsible for the radiocesium secondary contamination of surface waters on the contaminated areas for both accidents. Particulate and dissolved wash-off ratios in Chernobyl and Fukushima were found to decrease in the mid- and long term as a result of radiocesium depletion in the topsoil layer due to its vertical migration in catchment soils.

Particulate ^137^Cs wash-off ratios from the catchments of the Fukushima area display only minor differences compared with those in the Chernobyl area, being at the lower limit of the Chernobyl values. Somewhat lower values of *N_p_*(^137^Cs) in the Fukushima area are explained by higher values of the effective dispersion coefficient *D_eff_*(^137^Cs) in typical Fukushima soils. Dissolved ^137^Cs wash-off ratios for Fukushima catchments are at least an order-of-magnitude lower than those for Chernobyl mainly due to an order-of-magnitude difference in the ^137^Cs distribution coefficients for the Fukushima and Chernobyl rivers.

In summary, data resulting from Chernobyl long-term studies can be further used to refine predictions of temporal changes in the radionuclide behavior for Fukushima areas.

## Figures and Tables

**Table 1:** ^137^Cs apparent distribution coefficient *K_d_* (L/kg) in suspended sediment–water system of rivers in Chernobyl- and Fukushima-contaminated areas.

River-Site	Observation Period	Mean Value	References
Chernobyl			
Pripyat River-Chernobyl	1990–2016	(3.5 ± 0.6) × 10^4^	[18,102]
Dneper River-Nedanchichi	1989–2012	(6.4 ± 2.0) × 10^4^	[18,102]
Uzh River-Cherevach	1987–1990	(3.1 ± 2.0) × 10^4^	[47]
Fukushima			
Abukuma River-Kuroiwa	2012–2020	(6.5 ± 3.0) × 10^5^	[99]
Ukedo River downstream	2015–2018	(2.2 ± 0.3) × 10^5^	[97]
Ukedo River at Ogaki dam inflow	2014–2019	(6.3 ± 2.0) × 10^5^	[98]
Kodeya River at Ogaki dam inflow	2014–2019	(8.6 ± 2.1) × 10^5^	[98]
Ukedo River at Ogaki dam outflow	2014–2019	(4.5 ± 1.8) × 10^5^	[98]
Ohta River downstream	2015–2018	(2.4 ± 0.6) × 10^5^	[97]
Hiso River (Niida River system)	2011–2020	(4.6 ± 3.0) × 10^5^	[100]
Wariki River (Niida River System)	2011–2020	(7.7 ± 6.3) × 10^5^	[100]

**Table 2:** Typical values of mobile fraction at equilibrium $M_{\infty }$(^137^Cs) and diffusional fixation parameter δ(^137^Cs) for basic groups of soil types [68].

Group of Soil Types	$M_{\infty }$(^137^Cs), %	δ(^137^Cs), Day^1/2^
Sandy	14 ± 5	3 ± 2
Mineral	12 ± 8	10 ± 4
Turf	6 ± 5	50 ± 30

**Table 3:** Parameters used to approximate time dependence of particulate ^137^Cs in Pripyat River at Chernobyl and Dneper River at Nedanchichi by two-exponential model (Equation (12)).

River (Cross-Section)	Two-Exponential Model (Equation (12))			
	$C_{s1}^{0},$ Bq·g^−1^	$C_{s2}^{0},$ Bq·g^−1^	*k*_1_, Year^−1^	*k*_2_, Year^−1^
Pripyat (Chernobyl)	20	3.3	0.58	0.022
Dneper (Nedanchichi)	5.0	0.6	0.15	0.045

## Data Availability

This review article is based on previously published data.
